# The involvement of HDAC3 in the pathogenesis of lung injury and pulmonary fibrosis

**DOI:** 10.3389/fimmu.2024.1392145

**Published:** 2024-09-26

**Authors:** Hanming Yu, Shi Liu, Shuo Wang, Xiu Gu

**Affiliations:** Department of Pulmonary and Critical Care Medicine, The Fourth Affiliated Hospital of China Medical University, Shenyang, China

**Keywords:** histone deacetylase 3, acute lung injury, pulmonary fibrosis, inflammation, macrophage

## Abstract

Acute lung injury (ALI) and its severe counterpart, acute respiratory distress syndrome (ARDS), are critical respiratory conditions with high mortality rates due primarily to acute and intense pulmonary inflammation. Despite significant research advances, effective pharmacological treatments for ALI and ARDS remain unavailable, highlighting an urgent need for therapeutic innovation. Notably, idiopathic pulmonary fibrosis (IPF) is a chronic, progressive disease characterized by the irreversible progression of fibrosis, which is initiated by repeated damage to the alveolar epithelium and leads to excessive extracellular matrix deposition. This condition is further complicated by dysregulated tissue repair and fibroblast dysfunction, exacerbating tissue remodeling processes and promoting progression to terminal pulmonary fibrosis. Similar to that noted for ALI and ARDS, treatment options for IPF are currently limited, with no specific drug therapy providing a cure. Histone deacetylase 3 (HDAC3), a notable member of the HDAC family with four splice variants (HD3α, -β, -γ, and -δ), plays multiple roles. HDAC3 regulates gene transcription through histone acetylation and adjusts nonhistone proteins posttranslationally, affecting certain mitochondrial and cytoplasmic proteins. Given its unique structure, HDAC3 impacts various physiological processes, such as inflammation, apoptosis, mitochondrial homeostasis, and macrophage polarization. This article explores the intricate role of HDAC3 in ALI/ARDS and IPF and evaluates its therapeutic potential the treatment of these severe pulmonary conditions.

## 1 Introduction

Sepsis, a major global health threat, is predominantly characterized by high morbidity and mortality rates. An excessive inflammatory response induced by sepsis can precipitate multiorgan dysfunction and ultimately cause organ failure in severe cases (1). The lung is a particularly vulnerable organ to infection and the systemic inflammatory disorder sepsis (2). An immune and inflammatory response is evident within lung tissue during sepsis, impacting the integrity of the alveolar-capillary membrane barrier and contributing to edema formation (3). Due to the complexity of its underlying mechanisms, effective treatments for acute lung injury (ALI) remain elusive, with supportive care being the only available option to date.

ALI is an extreme clinicopathological entity characterized by unyielding hypoxemia and escalating dyspnea. In the absence of timely intervention, ALI has the potential to intensify to acute respiratory distress syndrome (ARDS) and multiple organ dysfunction syndrome (MODS). The latter two are important causes of death in critically ill patients (4–6). The central pathophysiological characteristics include enhanced pulmonary vascular permeability, interstitial edema, alveolar fibrin leakage, augmented intrapulmonary shunting, and a disruption in ventilation-perfusion equilibrium (7, 8). After acute inflammation and damage, the lung activates repair and remodeling processes to regain homeostasis. The resultant fibrosis and scarring potentially resolve over time or endure, accelerating persistent long-term pulmonary fibrotic damage (9–11). Signaling pathways act as crucial agents in the treatment of ALI and lung fibrosis. The initiation of these events by lipopolysaccharide (LPS) stimulation triggers several molecular intracellular signaling activities, including the classical activation of nuclear factor-kappa-B (NF-κB). This facilitates its translocation to the nucleus, consequently leading to the release of predominantly inflammatory cytokines, specifically interleukins (IL-1β, IL-2, and IL-6) and chemokines (macrophage inflammatory protein, MIP-1 α/β) (12–14). Within the scope of epigenetic mechanisms, strategic alterations in histone acetylation and deacetylation play a pivotal role in governing DNA accessibility (15). The deacetylation reaction is executed by histone deacetylases (HDACs), a series of enzymes partitioned into four classes (I-IV). Their primary function is to remove acetyl residues from histone tails, which facilitates DNA pairing with nucleosomes, prompts heterochromatin formulation, and hinders gene transcription (16). The activation and differentiation of fibroblasts into myofibroblasts, characterized by the expression of contractile proteins and an antiapoptotic phenotype, are critical factors involved in the pathogenesis of IPF (17, 18). The upregulation of HDAC in fibroblasts has been observed in IPF (19, 20). Furthermore, the use of HDAC inhibitors (HDACis) has demonstrated beneficial effects in preventing or reversing fibrogenesis (19–21).

IPF is an age-associated, progressively worsening, chronic, and ultimately lethal condition that manifests for several years and is characterized by inadequate remodeling of the lung parenchyma. The etiology of IPF is hypothesized to be centered on epithelial cell-mediated fibrosis. These cells are capable of activating fibroblasts that are responsible for extracellular matrix (ECM) components, leading to the fibrotic process (22, 23). IPF, a chronic and progressive fibrotic interstitial lung disease, remains elusive and is not entirely understood. Presumably, IPF is precipitated by repetitive harm to alveolar epithelial cells and consequent inadequate repair. The median survival duration postdiagnosis of IPF patients is projected to be between 2.5 and 3.5 years (22). Within the context of IPF, epigenetic reorientation occurs due to cell-microenvironment interactions, which establish a feedback loop that fosters a pro-fibrosis environment. This feedback loops subsequently bolsters the differentiation of cells into myofibroblasts and catalyzes the expression of genes implicated in the maladaptive restoration of the extracellular matrix (24–26). Presently, therapeutic options for IPF are notably limited, with lung transplantation remaining the only definitive treatment. The global prevalence of IPF is increasing, correlating with significant increases in morbidity and mortality. Moreover, this disease imposes a considerable economic burden on health care systems worldwide.

The roles of certain HDACs in ALI have been uncovered recently. For instance, in mice with pneumonia-triggered ALI, HDAC7 inhibition through trichostatin A significantly mitigates inflammatory damage in lung tissue, subsequently enhancing survival (27). The inhibition of histone deacetylase 3 (HDAC3) significantly curtails the mitochondrial pathway of apoptosis while preserving the mitochondrial membrane potential in cases of cold renal storage/transplantation injury (28). Furthermore, HDAC3 can regulate inflammatory reactions through its unconventional deacetylase-independent activity, thereby controlling the inflammatory response (29). HDAC3 has been shown to be a critical regulator necessary for the macrophage inflammatory response following stimulation by inflammatory factors (30). Additionally, HDAC3 affects phagocytosis in M2 phenotype macrophages by regulating the transforming growth factor-β (TGFβ) and HDAC3-associated Smad signaling pathways (31). Furthermore, HDAC3 plays a significant role in modulating the inflammatory response and endotoxin tolerance in human monocytes and macrophages (32). A recent study has demonstrated that the attenuation of HDAC3 significantly enhances survival in mice with LPS-induced endotoxemia (33).

Despite progress made in recent decades, an optimal therapeutic strategy for ALI has yet to be developed, and broadly targeted anti-inflammatory therapies have primarily been unsuccessful in improving mortality rates. This lack of progress is largely due to the underlying molecular mechanisms that drive ALI, which continue to be inadequately understood (8). On the other hand, the cause and pathogenesis of IPF are currently unclear. With patients having a grim prognosis and limited treatment options available, it has become fundamental to gain an in-depth understanding of the molecular mechanisms steering both ALI and IPF for the prospective management of these conditions. Epigenetic modifications, which influence gene expression and developmental pathways without altering the underlying DNA sequence, hold potential as targets for such novel interventions. Among the principal mechanisms of epigenetic regulation, modifications of histones, specifically through acetylation and deacetylation, have been implicated in an array of pathological and physiological processes within mammalian systems (34). Under various pathological conditions, epigenetic alterations of histones in eukaryotic cells are known to modulate gene expression by promoting chromatin structural stability, which can precipitate oxidative stress, inflammation, and cellular apoptosis (35, 36). HDAC3 exerts its regulatory role not only by deacetylating histones and thus modulating gene transcription but also by posttranslationally modifying a variety of nonhistone proteins, including select proteins within mitochondrial and cytoplasmic compartments (35). In this review, we outline the significance of HDAC3 and its inhibitors in the context of acute lung injury and pulmonary fibrosis, as observed in recent years, in an attempt to provide some clinical implications.

## 2 Biological properties of histone deacetylases

Histone deacetylases (HDACs) are a family of proteases that modify histone deacetylation, regulate chromatin state, and ultimately affect gene expression (37) and play an important role in epigenetic regulation. Eighteen different HDAC isoforms have been identified in mammals and are classified into four classes based on their homology and subcellular localization with yeast germline HDACs (38, 39). Class I HDACs include HDAC1, HDAC2, HDAC3 and HDAC8, the sequences of which are homologous to those of the yeast deacetylase reduced potassium dependency 3 (RPD3), which is mainly localized in the nucleus and involved in the regulation of cell proliferation (39, 40). Class II HDACs can be further divided into class IIa (HDAC4, HDAC5, HDAC7 and HDAC9) and class IIb (HDAC6 and HDAC10) (37, 41). Class III HDACs do not contain a zinc finger binding domain in their structure and are dependent on the metabolic cofactor nicotinamide adenine dinucleotide (NADD) (37, 42). The structure of class III HDACs does not contain a zinc finger binding domain, and these enzymes rely on the metabolic cofactor nicotinamide adenine dinucleotide (NAD+) for deacetylation. Class IV HDACs, localized in the nucleus and comprising solely HDAC11, possess a catalytic core region akin to that of Class I and Class II HDACs (43). HDAC11 has multiple functions, including the regulation of interleukin-10 (IL-10) expression (42) and the modulation of type I interferon signaling (44).

**Figure 1:**
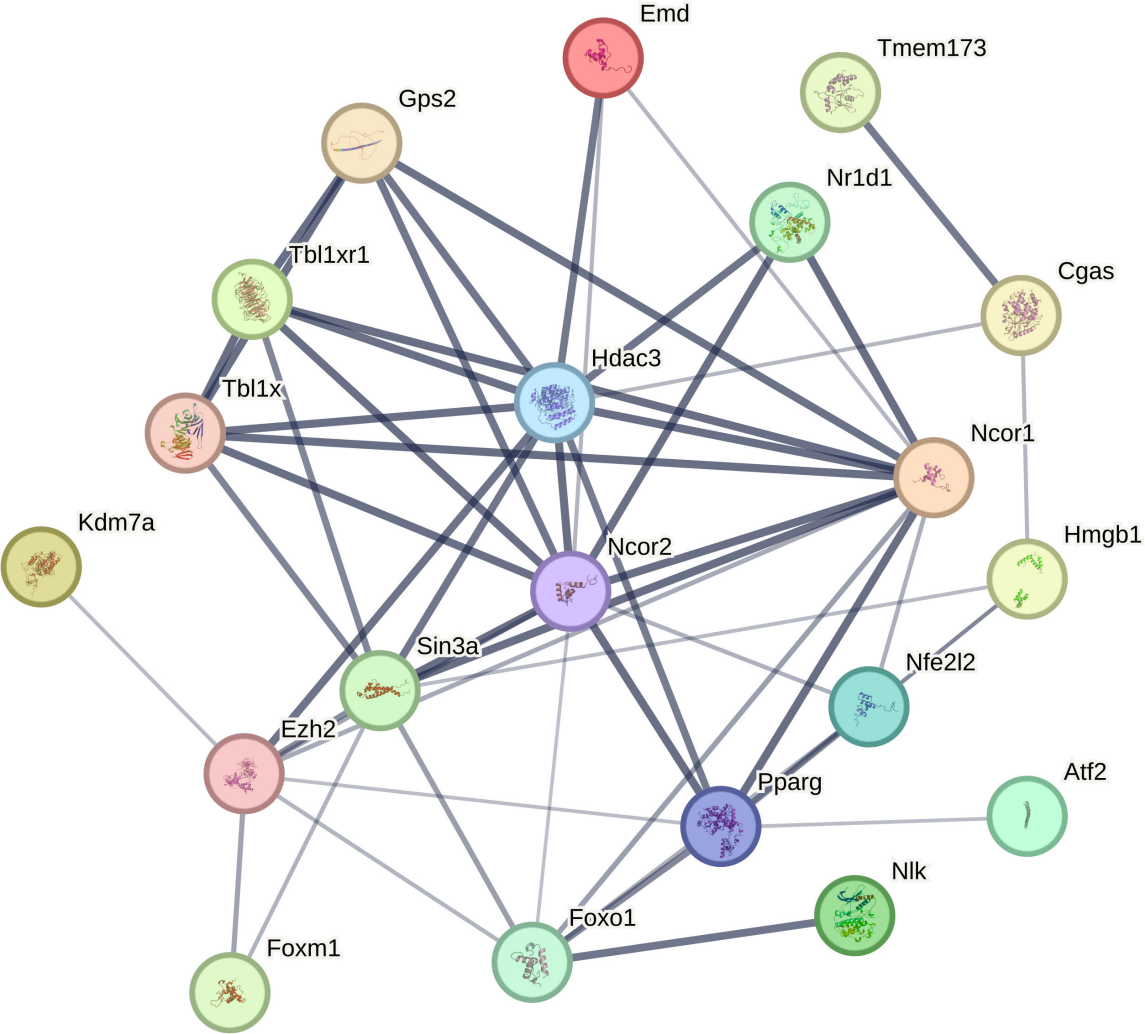
HDAC3 interacts directly or indirectly with a number of transcriptional regulatory proteins (from STRING.COM).

As a constituent of class I HDACs, HDAC3 is reported to be distinct within the HDAC chromatin modifier superfamily. In the nucleus, its activity differs with that of other deacetylases owing to its exclusive substrates. These include lysine 9 of histone 3 (H3K9ac) and lysine 5 (H3K5ac), along with lysine 9 of histone 4 (H4K9ac) (45, 46). HDAC3 plays a role in numerous inflammatory conditions, organ fibrosis, neurodegeneration, and ischemic injury. It accomplishes this by mediating histone deacetylation, subsequently fostering chromatin condensation and transcriptional repression (47). HDAC3 not only modulates gene transcription by deacetylating histones but also posttranslationally modifies nonhistone proteins, which include particular mitochondrial and cytoplasmic proteins (35, 36). Although HDAC3 is well-recognized for its deacetylation function, recent reports have demonstrated that HDAC3 can also act as an “eraser” of lactonylation (48, 49). Lactonylation, a novel epigenetic modification, plays a crucial role in macrophage activation and in the regulation of lung injury and fibrosis (50, 51). During the later stages of M1 macrophage polarization, increased histone lactylation induces the expression of homeostatic genes involved in wound healing, such as Arginase 1 (52). Recent studies have identified HDAC3 as a highly potent delactoylase, exhibiting activity levels thousands of times higher than SIRT2, previously considered the primary delactoylase *in vivo* (53). The de-L-lactamase activity of class I histone deacetylases (HDAC1-3) within cells has been further validated in a study by Moreno-Yruela et al. (49).HDAC3 either directly or indirectly interacts with various transcriptional regulatory proteins (**Figure 1**), suggesting that HDAC3 is involved in a diverse array of signaling pathways and cellular processes.

## 3 HDAC3 in acute lung injury

LPS stimulation significantly upregulated HDAC3 mRNA and protein levels in mouse lung tissues (33, 54, 55).

### 3.1 Impact of HDAC3 regulation on macrophage-driven lung inflammation

Macrophage cohorts in the lungs maintain homeostasis by phagocytizing inhaled particles and foreign pathogens, triggering cytokine production, and facilitating antigen presentation. These actions collectively contribute to the eradication of particulate antigens (56, 57). Although macrophages indeed play a pivotal role in fending off invading organisms, their overabundance may precipitate tissue damage (58). Within the context of sepsis-induced ALI, lung tissue experiences an influx of a significant number of peripheral macrophages. This infiltration results in the increased production of various proinflammatory cytokines, such as tumor necrosis factor alpha (TNF-α) and interleukin (IL)-1β. Additionally, increased release of high mobility group box 1 (HMGB1) is noted (12, 57). In subsequent processes, neutrophils in the circulatory system are mobilized toward the alveolar space and interstitial tissues, precipitating inflammatory exudation and tissue damage (57). When macrophages undergo LPS activation and in the absence of nuclear receptor corepressor 1/2 (NCoR1/2), HDAC3 encroaches upon the activating transcription factor 2 (ATF2) binding site, initiating the expression of inflammatory genes in a noncanonical fashion. Conversely, the deacetylase activity of HDAC3 selectively interacts with the ATF3 binding site, inhibiting Toll-like receptor (TLR) signaling. Interestingly, HDAC3 deletion in macrophages resulted in LPS resistance in mice (29).

A study led by Natoli and colleagues revealed that upon LPS stimulation, close to half of the inflammatory genes in macrophages were not expressed when HDAC3 was inhibited. This finding suggested that HDAC3 inhibition has a potential anti-inflammatory effect (59). HDAC3 is capable of initiating the cyclic GMP-AMP synthase (cGAS)–stimulator of interferon genes (STING) pathway by mitigating histone acetylation of the miR-4767 gene promoter, which is induced by LPS in macrophages. This trigger initiates an inflammatory response and thermokinetic-selectivity (33, 36). By modulating HDAC3 to suppress macrophage activation, the level of inflammation-related cytokines in the lung decreases, which subsequently improves the severity of LPS-induced septic shock. This process operates through the HMGB1/NF-κB axis (60). HDAC3 was identified as a critical modulator of IL-12b transcription. IL-10 reduces IL-12b synthesis by regulating HDAC3 activity on the IL-12b promoter. A notable reduction in the nuclear translocation of the p65 NF-κB subunit RelA in LPS-stimulated alveolar macrophages is observed with the disruption of the class I HDAC signaling axis by IL-10 (61).

### 3.2 Unraveling the role of HDAC3 in regulating the NF-κB pathway and inflammatory response

Several intracellular signaling events, including classic NF-κB activation, commence upon LPS stimulation. This action facilitates nuclear translocation, resulting in the release of primary inflammatory cytokines, chiefly interleukins (IL-1β, IL-2, and IL-6) and chemokines (macrophage inflammatory protein, MIP-1α/β) (12). In an extensive study, Elisabeth Ziesché and colleagues deciphered the pivotal role of HDAC-3, demonstrating its critical function as a coactivator in IL-1-induced inflammatory signaling, and this role is achieved through the removal of acetyls inhibited by p65NF-κB (62). Similarly, a study conducted by Leus and colleagues used RGFP966, an HDAC-3-selective inhibitor, in a lung inflammation model. They observed an increase in NF-κB transcription paired with a decrease in the expression of inflammatory cytokines, such as IL-1β, IL-6, and IL-12b, in macrophages. Concurrently, they noted an upregulation of the anti-inflammatory cytokine IL-10 (63). This notable study highlights the potential role of inhibiting NF-κB and HDAC-3 translocation. Such inhibition results in a reduction in proinflammatory cytokines and fosters redox homeostasis, thereby alleviating the inflammation typically associated with ARDS (55).

**Figure 2:**
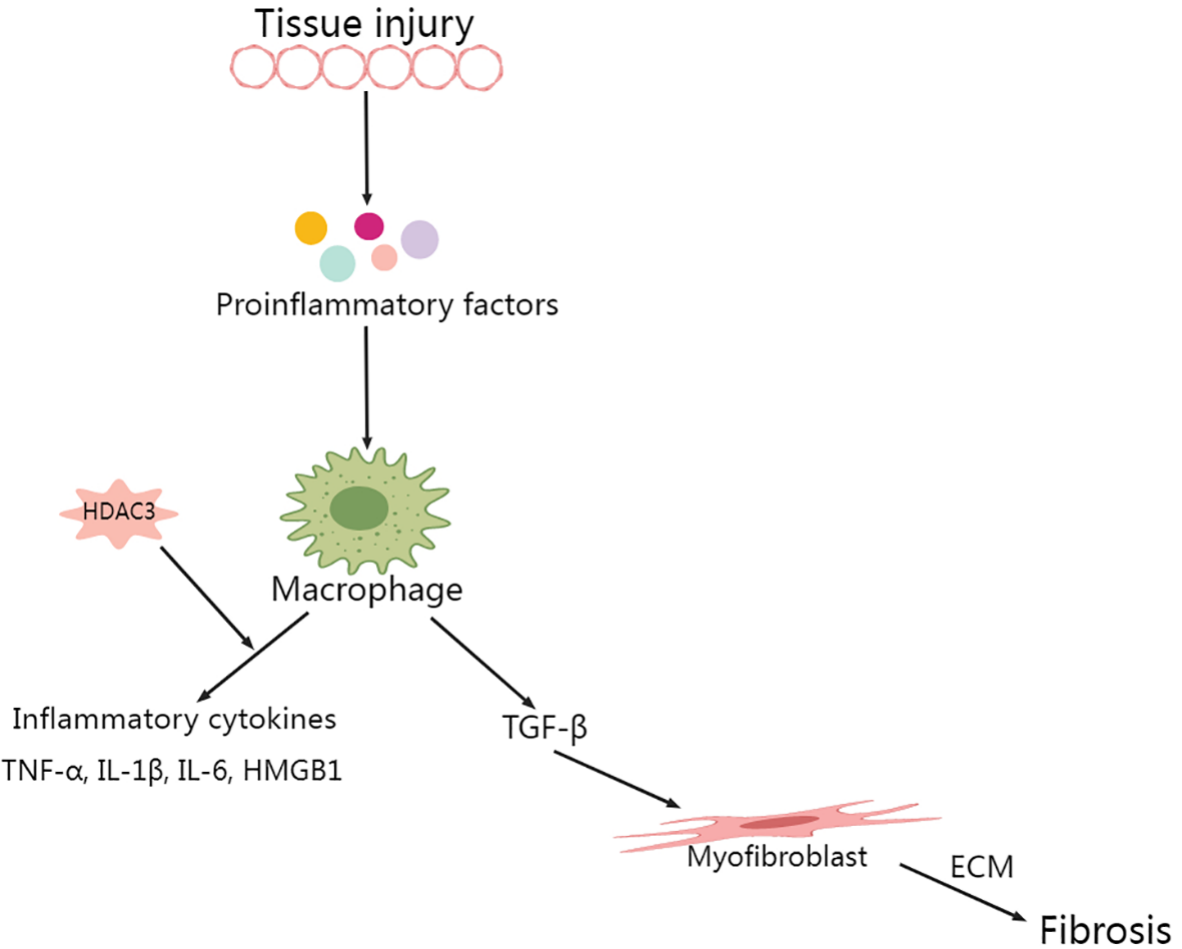
Role of HDAC3 in macrophage secretory spectrum during lung injury.

The significance of HDAC3 in promoting HMGB1 hyperacetylation and subsequent translocation is gaining recognition (60, 64, 65). Studies have shown that elevated HDAC3 levels inhibit LPS-induced HMGB1 translocation and secretion in RAW264.7 cells (65). Specifically, HDAC3 can enhance the transcriptional activity of the NF-κB p65 subunit and modulate the NF-κB signaling pathway through deacetylation, thereby reducing inflammatory responses (64). In *in vitro* experiments, HDAC3 reduces the acetylation level of HMGB1, inhibiting its translocation from the nucleus to the cytoplasm, a process associated with the intervention of NF-κB signaling (65). *In vivo* studies also show that the deletion or inhibition of HDAC3 leads to increased HMGB1 hyperacetylation, which can elevate the expression of inflammatory factors, indicating the crucial role of HDAC3 in regulating inflammatory responses (36). Moreover, *in vivo* research by Guo et al. corroborated the concept that HDAC3-dependent pathways may impair the nuclear factor erythroid 2-related factor 2 (Nrf2)/antioxidant response element (ARE) pathway through NF-κB activity, thereby exacerbating oxidative stress-induced necroptosis and ischemia/reperfusion injuries by facilitating the release of HMGB1 (66). Other studies have also demonstrated a key regulatory role for HDAC in HMGB1 translocation (55, 67, 68). Wen Q and colleagues demonstrated that HDAC3 modulation mitigated LPS-induced septic shock via the HMGB1/NF-κB pathway (60). On the other hand, HDAC3 suppression resulted in decreased TNF-α levels, which were accompanied by a correlated increase in acetylated p65. Notably, p65 is an NF-κB subunit that has been reported to play a significant role in inhibiting IκB-mediated NF-κB transcriptional activity (**Figure 2**) (69, 70).

### 3.3 HDAC3 modulates mitochondrial balance, fatty acid metabolism, and epithelial disruption

Mitochondria, characterized by an elongated dual-membrane structure, reside in the cytoplasm of virtually all eukaryotic cells and possess a unique self-replicating genome (71). Wang XR and colleagues reported that mitoquinone (MitoQ) mitigated alveolar epithelial cell apoptosis and prevented barrier disintegration by controlling mitochondrial fission during sepsis-induced ALI (72).

**Figure 3:**
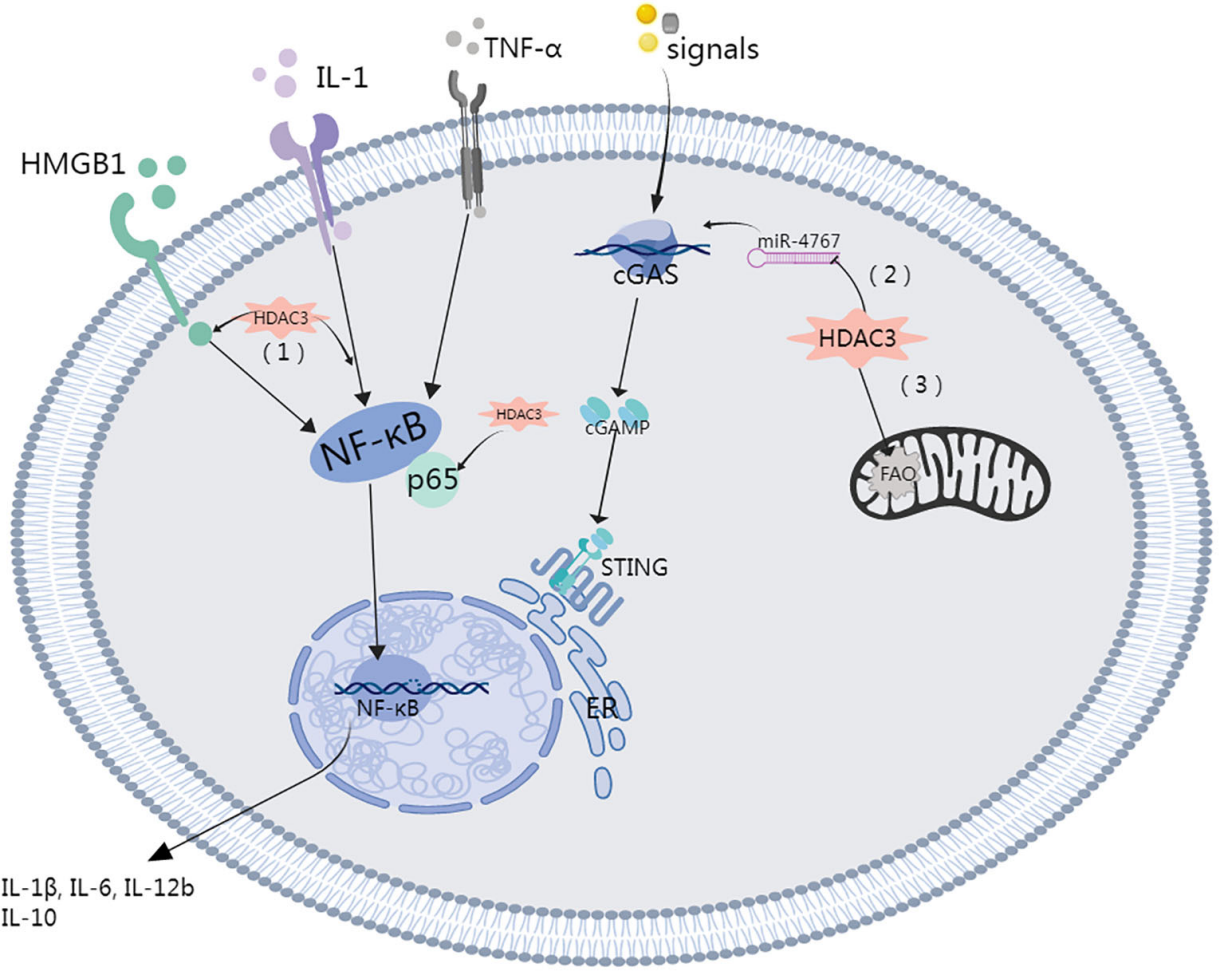
Role of HDAC3 in the lung injury.

HDAC3 facilitates the regulation of gene transcription through the deacetylation of histones but also posttranslationally modifies nonhistone proteins, notably certain cytoplasmic and mitochondrial proteins (35). A recent study highlighted the ability of HDAC3 to migrate to mitochondria, where its deacetylates an enzyme related to fatty acid oxidation (FAO), specifically the α-subunit of the mitochondrial trifunctional enzyme. This activity effectively impedes FAO within macrophages (73). RGFP966-mediated HDAC3 inhibition enhances mitochondrial membrane potential and diminish mitochondria-mediated apoptosis (28). Likewise, in scenarios involving diabetes and obesity, the pharmacological suppression of class I HDACs, accomplished through agents such as SAHA or MS275, augments mitochondrial function and oxidative capacity in adipose tissue and skeletal muscle (74). Indeed, HDAC3 plays a crucial role in energy metabolism and the maintenance of mitochondrial equilibrium. Remarkably, a significant decrease in the expression of genes related to mitochondrial oxidative phosphorylation was observed in brown adipose tissue deficient in HDAC3, resulting in diminished mitochondrial respiration. These findings suggest that HDAC3 plays an indispensable role in thermogenesis within brown adipose tissue (75). HDAC3 deficiency in brown adipose tissue resulted in considerable downregulation of mitochondrial oxidative phosphorylation genes, resulting in diminished mitochondrial respiration. This finding illustrates the crucial role of HDAC3 in thermogenesis within brown adipose tissue. Furthermore, HDAC3 facilitates Rho-associated, coiled-coil containing protein kinase 1 (ROCK1) transcription and activation via the deacetylation of Forkhead box O1 (FOXO1), which subsequently impairs mitochondrial quality control (MQC) in alveolar epithelial type II cell (AT2). This chain of events ultimately culminates in damage to the epithelial barrier and ALI (**Figure 3**) (54, 76).

## 4 HDAC3 in pulmonary fibrosis

The augmentation of HDAC3 is observed in diverse lung fibrosis models (77, 78), and HDAC3 is predominantly located within fibrotic lesions and pulmonary fibroblasts (79–81).

The formation of myofibroblasts from the epithelial–mesenchymal transition (EMT) of alveolar epithelial cells is a critical event in IPF (82, 83). Myofibroblasts not only are capable of synthesizing substantial amounts of ECM but also exhibit smooth muscle cell-like characteristics, such as the expression of α-smooth muscle actin (α-SMA) (84). These traits endow myofibroblasts with contractile abilities, further contributing to tissue stiffness and airway constriction (84, 85).

Lung tissue hypoxia is intrinsically linked to inflammation and fibrosis (86–88). In their seminal work, Zhou et al. elucidated how hypoxia triggers EMT under the influence of mitochondrial reactive oxygen species and hypoxia-inducible factor-1 (HIF-1) (89). Similarly, Higgins et al. revealed that hypoxia induces EMT via HIF-1 activation, ultimately promoting fibrogenesis (90). HDAC3 promotes the alveolar epithelial-mesenchymal transition and fibroblast migration under hypoxic conditions (91). This pathway directly enhances fibroblast migration and invasion. Intriguingly, a separate study implicated TGF-β1/SMAD3 signaling pathway activation as the direct trigger of the heightened HDAC3 expression typically observed in pulmonary fibrosis (92). Of paramount significance is the role of HDAC3 in mediating EMT in AT2 cells and pulmonary fibrosis. This is achieved through GATA3 deacetylation, which subsequently prevents its degradation (92). Additionally, miR-224 plays a crucial role in facilitating the HDAC3-induced EMT, primarily through the suppression of mRNA translation for recombinant forkhead box A1 (FOXA1) (91).

Pulmonary fibrosis is distinctively characterized by an increase in matrix stiffness, a consequence of the accumulation of ECM. This effectively established a self-propagating cycle that is favorable for fibroblast proliferation (93, 94). In this context, HDAC3 has been identified as a key player in the mechanotransduction response mechanism. Specifically, it contributes to regulating changes in cell morphology, chromatin accessibility, hyperacetylation, and gene expression that are typically driven by matrix stiffness or geometrical cellular constraints (95–97). HDAC3 is essential for preserving the acetylation-to-methylation ratio of H3K9 residues (98, 99). The absence of nuclear HDAC3 results in increased acetylation and decreased expression of both heterochromatin markers (H3K9me2) and fibronectin (100).

Recent research underscores the significant role of HDAC3 in the progression of fibrotic lung disease. This work indicated that RGFP966, a selective HDAC3 inhibitor, effectively diminished lung fibrosis in wild-type mice subjected to bleomycin treatment. Conversely, Hdac3 KO mice exhibit a high degree of resistance to bleomycin-induced lung fibrosis (79). Moreover, it was determined that IPF lungs exhibited HDAC3 overexpression on Days 14 and 21 after bleomycin treatment, which coincided with suppressed Nrf2 expression and expression of the pro-fibrosis forkhead box M1 (FOXM1) transcription factor during lung fibrosis progression. In contrast, RGFP966 therapy reduced HDAC3 and FOXM1 binding and increased the expression of acetylated histone H3 at the Nrf2 promoter site. These effects subsequently increased Nrf2 repression and concurrently increased the expression of Nrf2-induced antioxidants. In addition, this treatment restored epithelial E-calmodulin expression and decreased fibronectin expression (79). Similarly, Nrf2 expression was markedly absent in the fibroblast foci of IPF patients (101), whereas HDAC3 expression increased (19). It was determined that HDAC3 expedites the progression of pulmonary fibrosis by stimulating EMT and inflammation via the Notch1 or signal transducer and activator of transcription 1 (STAT1) signaling pathways (102). In summary, the atypical excessive expression of HDAC3 coupled with its inhibition of Nrf2 plays a pivotal role in IPF. Importantly, this can be moderated through the selective inhibition of HDAC3.

**Figure 4:**
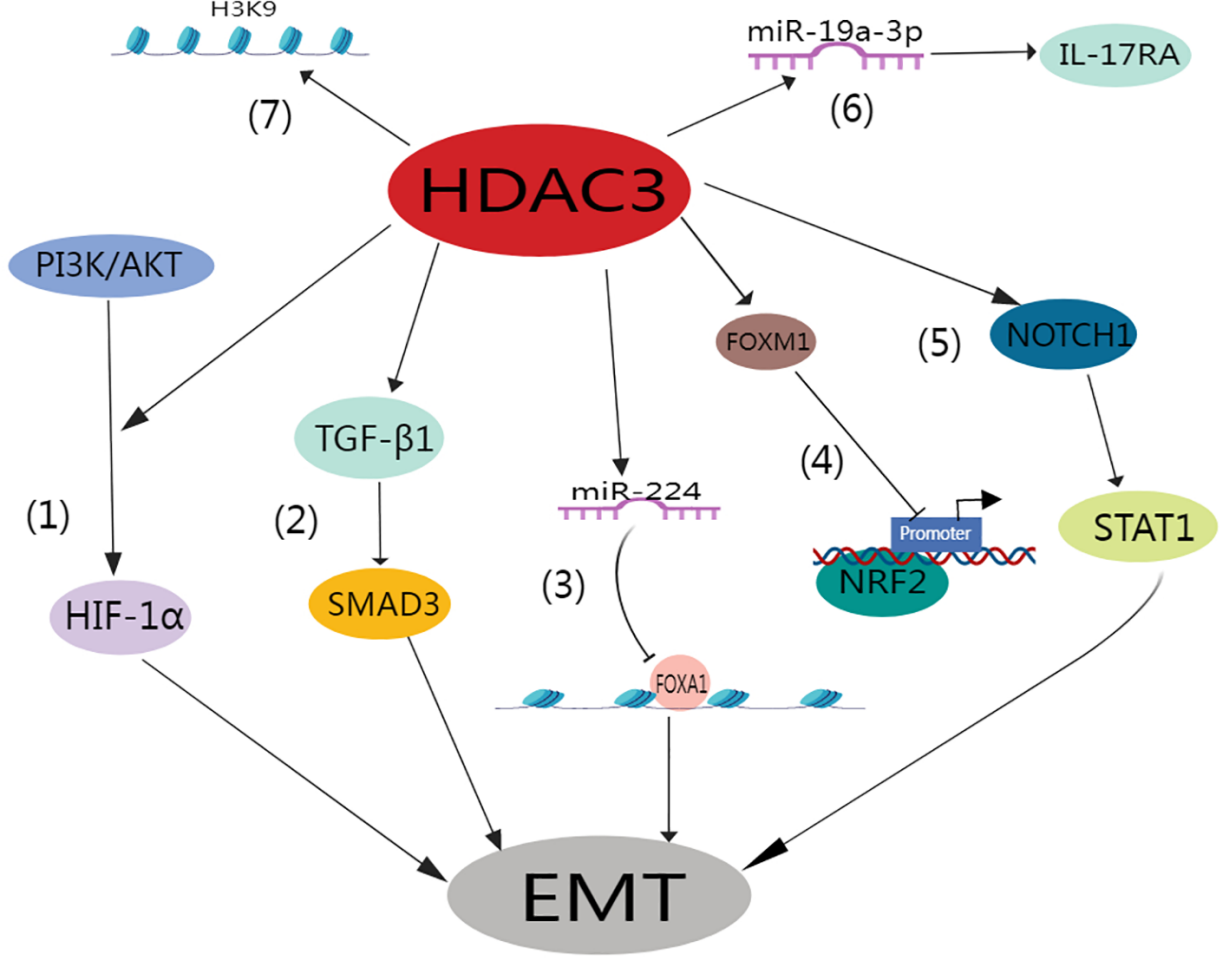
Role of HDAC3 in the pulmonary fibrosis.

Moreover, HDAC3 increases interleukin 17 receptor A (IL17RA) expression in rheumatoid arthritis (RA)-associated interstitial lung disease (ILD) by down-regulating miR-19a-3p (81). Intriguingly, HDAC3 and IL17RA levels were elevated in the lung tissues of individuals with RA-ILD compared to healthy controls. Deeper examination revealed a positive correlation between the expression of HDAC3 and that of IL17RA in RA-ILD patients. Using a mouse model of RA-ILD, it was determined that HDAC3 downregulated the expression of miR-19a-3p in the lung fibroblasts of these mice, subsequently enhancing IL17/IL17RA signaling and ECM protein expression. However, this impact could be nullified through overexpression of miR-19a-3p or by silencing Il17ra or Hdac3 using siRNAs. Notably, the administration of Hdac3-targeted siRNA to RA-ILD mice noticeably ameliorated pulmonary fibrosis *in vivo* (**Figure 4**) (81).

## 5 Potential role of HDAC3 in ALI and IPF through NLRP3 activation

### 5.1 Role of HDAC3 in NLRP3 inflammasome initiation

The activation of NF-κB, which is promoted by primary signals, facilitates NLRP3 (NOD-, LRR-, and pyrin domain-containing (3)), pro-IL-1β, and pro-IL-18 transcription (103). The NF-κB transcription factor complex is at the epicenter of inflammatory gene regulation in response to the onset of immune and inflammatory stimuli. Notably, the acetylation status of p65, a subunit of NF-κB, has been implicated in its nuclear translocation (69, 104). Ziesché E et al. reported that HDAC3 is extensively coactivated via the deacetylation of NF-κB p65 and that the ablation of HDAC3 propels the acetylation of p65 (62). In LPS-stimulated mouse small glioma cells (BV2), HDAC3 inhibition correspondingly reduces the nuclear presence of p65, thereby curtailing NLRP3 protein expression and inflammation (105). These findings indicate that HDAC3 modulates p65 acetylation, thus amplifying the initiation of the NLRP3 inflammasome.

Furthermore, HDAC3 promotes NF-κB expression to increase pro-IL-1β transcription, IL-1β release, and inflammation via upstream NF-κB activators, including molecule myeloid differentiation factor 88 (Myd88), α-tubulin, and reactive oxygen species (ROS) (12, 62, 106–112). Documented interactions between HDAC3 and the TLR adapter molecule Myd88 suggest a modulatory influence (108, 111). A deficiency in HDAC3 seems to attenuate NF-κB induction through TLR4 stimulation and diminishes the secretion of inflammatory mediators, including IL-1β, IL-6, and TNF-α (108). HDAC3 inhibition increases the acetylation of α-tubulin, thus limiting microtubule depolymerization (109). ROS, primarily sourced from Nicotinamide Adenine Dinucleotide Phosphate (NADPH) oxidase (113, 114), comprises two membrane-bound subunits (p22phox and gp91phox), three cytosolic counterparts (p67phox, p47phox, and p40phox), and the small G protein Rac (Rac1 and Rac2) (115). HDAC3 upregulates the expression of Nox4-based NADPH oxidase subunits, augmenting ROS production (106, 110, 112). Hence, HDAC3 can promote NF-κB activation through Myd88, microtubules, or ROS, culminating in the engagement of the NLRP3 inflammasome; however, further elucidation of these mechanisms is imperative.

### 5.2 The role of HDAC3 in PPARγ/NLRP3 inflammasome activation

The peroxisome proliferator-activated receptor γ (PPARγ), a ligand-sensitive nuclear receptor, is regulated by posttranslational modifications, including phosphorylation, hemoylation, and ubiquitination (116). Recently, HDAC3 inhibition has been linked to increased acetylation and subsequent activation of PPARγ (117, 118) as well as elevated expression of PPARγ target genes (116). PPARγ activation can mitigate neuroinflammation by impeding the NLRP3 signaling cascade (119–122). Recent studies have shown that HDAC3 inhibition may attenuate neuroinflammation and enhance neurological outcomes by activating the PPARγ/NLRP3/GSDMD signaling pathway (120).

PPARγ can attenuate lung injury by inhibiting the activity of NLRP3 inflammatory vesicles through its anti-inflammatory effects (123, 124). Salvianolate Acid A (SA) efficaciously enhances the state of lipopolysaccharide-triggered acute lung injury in mice. This improvement is brought about by the diminution of M1 macrophage levels via the PPAR-γ/NLRP3 pathway (125). The activation of PPARγ, which targets the NLRP3/GSDMD/caspase-1 axis, by SYG has demonstrated potential for inhibiting macrophage pyroptosis (123). Another study showed that ACT001 (an anti-inflammatory agent) remarkably mitigates LPS-triggered acute lung injury. This occurs mainly through the modulation of the PPAR-γ/NLRP3/NF-κB signaling pathway embedded within macrophages (126). Vinpocetine activates PPAR-γ and down-regulates the NLRP3/NF-κB pathway, showing significant attenuation in lung fibrosis *in vivo* and *in vitro* (127). Moreover, in a murine model of acute liver failure, NLRP3 inflammasome-mediated inflammation and LPS-induced macrophage M1 polarization were ameliorated by modulating PPAR-γ methylation (128), which is consistent with the findings of Fu et al. (126).

The inhibitory effect of HDAC3 on NLRP3 inflammasome activation via PPARγ acetylation and activity enhancement is evident. Nevertheless, direct evidence in inflammation-afflicted macrophages remains elusive, and the current body of evidence suggests that the interplay between HDAC3 and PPARγ/NLRP3 may hold significance for treating inflammatory conditions by targeting macrophages.

### 5.3 Potential interplay of HDAC3 and NLRP3 in IPF pathogenesis

Recent studies have shown that in IPF patients, the inflammasome NLRP3 undergoes hyperactivation, contributing to an increase in the production of class I interleukins and collagen (129). The IL-1 superfamily is a group of 11 cytokines(IL-1α、IL-1β、IL-36α、IL-36β、IL-36γ、IL-36Ra、IL37、IL-38、IL-1Ra、IL-18和IL-33) that play a central role in the regulation of immune and inflammatory processes in response to a large group of stimuli (130). IL-18 and IL-18Ra expression is increased in patients with IPF, and experimental studies in mice have shown that administration of IL-18 exacerbates the progressive deposition of extracellular matrix components and the development of fibrosis (131). Within the lungs, IL-1β plays a crucial role in driving inflammation, promoting white blood cell migration, and contributing to the degradation of elastin fibers and the deposition of collagen (132). These pathological effects are, in part, mediated through the IL-17A signaling pathway (133, 134). These findings underscore the pivotal role of NLRP3 in fostering chronic lung injury and fibrosis (129). Furthermore, an investigation conducted by Giannarakis et al. (135) revealed a pronounced activation profile of the NLRP3 inflammasome in IPF, highlighting its significance in the pathological landscape of the disease.

Scutellaria (the principal active constituent in brevicaffin) has been demonstrated to mitigate inflammation and epithelial–mesenchymal transition (EMT) in bleomycin (BLM)-induced pulmonary fibrosis via the NF-κB/NLRP3 signaling axis (136). Concurrently, lycorine ameliorates BLM-induced pulmonary fibrosis by obstructing NLRP3 inflammasome activation and pyroptosis by targeting the pyrin domain (PYD) of the CARD (ASC) (137). These findings substantiate the integral role of the NLRP3 inflammasome in pulmonary fibrosis, indicating that certain anti-inflammatory therapeutics can modulate the disease course by inhibiting NLRP3 inflammasome activation, thereby attenuating pyroptosis.

Moreover, the activation of discoidin domain receptor 1 (DDR1) in macrophages has been identified as a proponent of IPF through the modulation of the NLRP3 inflammasome and macrophage responses (138). Similarly, SS-31 has been shown to ameliorate lung fibrosis and inflammation by inhibiting Nrf2-mediated NLRP3 inflammasome activation in macrophages (139). This insight provides a basis for future investigations into the interplay between macrophage-mediated HDAC3 and NLRP3 activation in the context of inflammation and fibrosis.

## 6 HDAC3 as a therapeutic target

HDAC3 is considered a potential therapeutic target for lung injury and pulmonary fibrosis. Studies have shown that HDAC3 is involved in the regulation of various biological processes, including inflammatory responses (102), immune cell function (140), cell survival and apoptosis (21, 79), and cell proliferation (54). All of these processes are associated with the onset and progression of lung injury and pulmonary fibrosis.

HDAC3 impacts various cellular signaling pathways, such as activating NOTCH1 and STAT1 signaling pathways, which influence macrophage activation and inflammatory responses from multiple angles (102). Additionally, research indicates that HDAC3 plays a crucial role in maintaining epithelial cell barrier integrity and mitochondrial quality control, particularly important in preventing acute lung injury (54). HDAC3 inhibitors have also shown potential in reducing pulmonary fibrosis by inducing fibroblast apoptosis and thus alleviating fibrosis (21, 92). Targeting HDAC3 specifically could provide a new treatment strategy for fibrosis, potentially improving therapeutic outcomes when combined with existing treatment methods (20, 91).

HDAC3 inhibitors may involve broad gene regulation, posing potential risks of side effects and toxicity, necessitating further research to confirm their safety (141). Additionally, current HDAC inhibitors lack target specificity, affecting other members of the HDAC family, which may lead to non-specific effects and side effects (79). Moreover, most research is still in the preclinical and animal experiment stage, lacking large-scale clinical trial data to confirm specific therapeutic effects and safety (140). Long-term use of HDAC3 inhibitors could also lead to drug resistance, which must be closely monitored in clinical applications and addressed through strategic planning (19).

Currently, the development of drugs that target HDAC3 involves a number of HDAC inhibitors; however, these agents tend to act on multiple HDAC isoforms rather than specifically targeting HDAC3. When developing therapeutics, establishing specific inhibitors is crucial for minimizing potential side effects and enhancing treatment efficacy.

## 7 Conclusion

Currently, the etiology and pathogenesis of ALI and IPF remain elusive, with patients often facing a dire prognosis and limited therapeutic options. HDAC3 inhibitors have emerged as potential pivotal agents for the treatment of acute lung injury and fibrotic diseases. HDAC3 influences the expression of inflammatory genes in macrophages, modulates the NF-κB signaling pathway, and affects the posttranslational modification of nonhistone proteins; these processes are crucial for the development and progression of lung injury and pulmonary fibrosis. Nonetheless, the precise mechanisms of action necessitate validation through extensive research in animal models of acute lung injury and pulmonary fibrosis as well as *in vitro* patient-derived studies. Therefore, targeting HDAC3 potentially represents an innovative therapeutic approach for acute lung injuries and fibrotic lung diseases. In summary, although research on the use of HDAC3 inhibitors for the treatment of lung injury and fibrotic diseases is in its infancy, with most studies being preclinical in nature, the preliminary evidence suggests that HDAC3 inhibitors have beneficial effects. However, translating these findings into clinical practice will require a substantial body of both basic and clinical research.
